# Hospitalization of elderly diabetic patients: characteristics, reasons for admission, and gender differences

**DOI:** 10.1186/s12877-016-0333-z

**Published:** 2016-09-05

**Authors:** Wei Lin, Chan Chen, Huaqin Guan, Xiaohong Du, Junjian Li

**Affiliations:** 1 Department of Geriatrics, First Affiliated Hospital of Wenzhou Medical University, Wenzhou, China; 2 Science and Technology Information Center, Wenzhou Medical University Library, Wenzhou, China; 3 Department of Gastroenterology, First Affiliated Hospital of Wenzhou Medical University, Wenzhou, China; 4 Department of Hepatobiliary and Pancreatic Surgery, First Affiliated Hospital of Wenzhou Medical University, Wenzhou, China

**Keywords:** Elderly, Diabetes mellitus, Hospitalization, Gender difference

## Abstract

**Background:**

Understanding the differences in characteristics, gender, and common causes for admission in hospitalized elderly diabetic patients provides a theoretical basis for their successful management. This study explored the reasons and gender differences in hospitalizations of elderly patients with diabetes mellitus.

**Methods:**

Patients aged ≥60 years who had received a diagnosis of diabetes by the time of discharge, from 1 January 2011 to 1 January 2014, were retrospectively enrolled. Hospitalization data of the patients were collected, and reasons for hospitalization were analyzed based on chief complaints and principle diagnosis.

**Results:**

The most frequent reasons stated for admission were related to the chronic complications of diabetes (42.1 %), seconded by hyperglycemia (26.4 %) and infection (15.7 %). Ketonuria, ketonemia, or diabetic ketoacidosis was more commonly seen in women than men, whereas diabetic nephropathy and neoplasms were more frequently found in men than women. Regarding infection as a cause of hospitalization, the 4 main types were respiratory tract (44.5 %), urinary tract (20.3 %), gastrointestinal (14.8 %), and skin and soft tissue (10.9 %). Respiratory tract infection was significantly more common in men (61.4 %) than women (31 %, *P* = 0.001), whereas urinary tract infection was more frequent in women (29.6 %) than men (8.8 %, *P* = 0.004).

**Conclusion:**

The most frequent reasons for hospital admission in elderly diabetic patients were chronic complications of diabetes, hyperglycemia, and infection. Men and women differed in reasons for hospital admission.

## Background

Diabetes mellitus is a worldwide epidemic whose prevalence is increasing rapidly. In China, 92.4 million adults are affected [1–3]. In particular, the population in China afflicted with this disease has ballooned in the last decade, causing expenditures of about 13 % of the nation’s health expenses [1–5]. Thus, diabetes mellitus is a serious challenge to health services in China.

The risk of diabetes and its associated complications rises with age, and these result in substantial morbidity and mortality in the elderly [6, 7]. In China, 20.4 % of those older than 60 years have diabetes [3]. While all persons with diabetes are at increased risk of hospitalization, the elderly in particular suffer from chronic complications and comorbidities [8], and may be hospitalized 2 or more times annually [9].

Due to a rapidly increased admission rate in the elderly with diabetes in recent years, it is important to investigate the frequency and reasons for their hospital admission. Indeed, there have been several studies reported in Chinese that investigate admission causes among diabetic patients in China [10–12]. However, most of them explore diabetic patients of all ages, including children, adults and elderly patients [10, 11], and the conclusions may vary depending on different research objectives. Little is known about the hospitalization of elderly patients with diabetes, especially in China. The elderly are a population with special features, such as functional and cognitive disabilities to various degrees and underlying co-morbidities, which require particular attention and more research. Understanding the differences in characteristics, gender, and common causes for admission in hospitalized elderly diabetic patients provides a theoretical basis for the successful management of such patients, and is also highly valuable for policy makers and hospital administrators to plan future health care services.

This study investigated the differences in characteristics, gender, and common causes for admission in hospitalized elderly patients with diabetes mellitus.

## Methods

### Study setting and patients

This retrospective cohort study consisted of patients aged ≥60 years who had received a diagnosis of diabetes, or its complications, by the time of discharge from First Affiliated Hospital of Wenzhou Medical College, China from 1 January 2011 to 1 January 2014. The patients who did not have a clear discharge diagnosis were excluded from this study. For patients who were admitted more than once, only the first admission was included. The study was approved by the Ethics Committee of First Affiliated Hospital of Wenzhou Medical University, China.

### Data collection

The reasons for hospitalization were collected based on the chief complaints of the patients and the main diagnosis at hospital discharge. When chief complaints differed from the discharge diagnosis, the discharge diagnosis was adopted. For the patients with more than one admission during this time, only the data of the first hospital admission were analyzed. The data from the electronic registration systems were collected and double-checked by two investigators. The clinical characteristics of the elderly diabetic patients that were collected included: age; gender; hospital length of stay; duration of diabetes; the time of diagnosis of diabetes mellitus; the reasons for admission; body mass index; waist-to-hip ratio; cardio-cerebrovascular complications; and chronic complications specific to diabetes including ketoacidosis, nephropathy, retinopathy, neuropathy, and peripheral arterial disease. The diabetes-related laboratory data were also recorded: fasting blood glucose and 2-h postprandial glucose (both at admission and at discharge), and hemoglobin A1c (HbA1c) level.

Subjects were eligible if they met the 1999 World Health Organization diagnostic criteria for diabetes mellitus [13] and were 60 years old or older. Arterial hypertension was defined as systolic blood pressure ≥ 140 mmHg or diastolic blood pressure ≥ 90 mmHg, or self-reported current treatment for arterial hypertension with antihypertensive medication. Retinopathy was diagnosed by ophthalmoscopy or retinal photography. Nephropathy was defined as the presence of microalbuminuria, gross proteinuria, or elevated serum creatinine level, in accordance with statements of the American Diabetes Association [14].

Coronary artery disease was established on the basis of documented events recorded by a physician, which could be angina, previous myocardial infarct, or coronary artery bypass graft or any other invasive procedures to treat coronary artery diseases. Peripheral artery disease was considered conditions such as ischemic foot ulcers, gangrene, amputation, vascular surgery, intermittent claudication, absent foot pulses, or abnormities identified by a Doppler ultrasound scan from clinical records.

### Statistical analysis

Data management and analysis were conducted using SPSS for Windows 17.0. Categorical variables are shown as percentages, whereas continuous variables are shown as mean ± standard deviation, except that hospital length of stay and age were reported as median and interquartile range (IQR) because of their skewed distribution. The chi-squared test was used to evaluate inter-group differences for categorical data, whereas the *t*-test was used for continuous data. *P*-values < 0.05 were considered statistically significant.

## Result

### General characteristics of patients

During the 3-year study period, 817 elderly diabetic patients, 402 women (49.2 %) and 415 men (50.8 %), were admitted to the hospital a total of 817 times. Among those hospitalizations, 89.2 % of the patients were aged 60–79 years, and 10.8 % were ≥80 years.

There were no significant differences between the men and women patients with regard to age, length of hospital stay, 2-h postprandial glucose at admission or discharge, HbA1c level, body mass index, or waist-to-hip ratio (Table 1). Female patients had significantly higher fasting blood glucose at admission, but lower fasting blood glucose at discharge, than did the men.

**Table 1:** Clinical characteristics of elderly diabetic patients ^a^

	Men	Women	*P*	Men/women, n/n
Age, y	70 (66–76)^b^	71 (66–76)^b^	0.747	415/402
Hospital stay, d	14.0 (10–19)^b^	14.0 (10–20)^b^	0.950	415/402
Fasting blood glucose at admission, mmol/L	8.49 ± 3.68	9.27 ± 4.97	0.013^c^	393/386
Two-hour postprandial glucose at admission, mmol/L	15.28 ± 5.76	15.37 ± 5.98	0.368	385/384
Fasting blood glucose at discharge, mmol/L	6.94 ± 2.20	6.57 ± 1.66	0.001^d^	369/377
Two-hour postprandial glucose at discharge, mmol/L	10.47 ± 2.91	10.68 ± 3.13	0.174	368/378
HbA1c level, %	8.14 ± 2.66	8.25 ± 2.69	0.445	310/331
Body mass index, kg/m^2^	23.92 ± 3.24	23.55 ± 3.59	0.197	310/316
Waist-to-hip ratio	0.94 ± 0.09	0.93 ± 0.09	0.568	300/313

^a^Mean ± standard deviation, unless stated otherwise

^b^median (IQR)

^c^ *P* < 0.05; ^d^ *P* < 0.01

Of the 817 admissions, 65 (8 %) received new diagnoses of diabetes and 752 (92 %) had a history of diabetes (10.88 ± 7.28 y). The prevalence of hypertension in the admitted patients was 64.4 % (526 cases). The HbA1c values were available for 641 (78.5 %) patients and blood glucose data were available for 746 (91.3 %) patients.

### Common reasons for hospital admission of elderly diabetic patients

The most frequent reason for admission of these elderly diabetic patients was chronic complications of diabetes (42.1 %; Table 2). Men and women had similar frequencies of chronic complications of diabetes (men, 42.4 %; women 41.8 %; *P* > 0.05). The second most frequent reason for admission was hyperglycemia or poor hyperglycemic control, with or without symptoms, which accounted for 26.4 % (216) of all admissions; the genders were similar (men, 28 %; women 24.9 %; *P* >0.05). Of the 216 patients admitted for hyperglycemia or poor hyperglycemic control, 47 (21.8 %) were asymptomatic, and 79 (36.6 %) presented with atypical symptoms such as weight loss, fatigue, blurred vision, and nocturia. The third most frequent reason for admission was infection, responsible for 15.7 % of hospitalizations.

**Table 2:** Common reasons for admission of elderly diabetics by gender ^a^

		Men	Women	Total
Subjects, *n*		415	402	817
Chronic complications of diabetes:		176 (42.4 %)	168 (41.8 %)	344 (42.1 %)
	Diabetic nephropathy	62 (14.9 %)	37 (9.2 %)^b^	99 (12.1 %)
	Diabetic retinopathy	19 (4.6 %)	36 (9.0 %)	55 (6.7 %)
	Diabetic neuropathy	39 (9.4 %)	45 (11.2 %)	84 (10.3 %)
	Peripheral arterial disease	21 (5.1 %)	12 (3.0 %)	33 (4.0 %)
	Cardio-cerebrovascular	35 (8.4 %)	38 (9.5 %)	73 (9.0 %)
Hyperglycemia		116 (28 %)	100 (24.9 %)	216 (26.4 %)
Infectious		57 (13.7 %)	71 (17.7 %)	128 (15.7 %)
Hypoglycemia		13 (3.1 %)	11 (2.7 %)	24 (2.9 %)
Neoplasms		17 (4.1 %)	6 (1.5 %)^b^	23 (2.8 %)
Ketonuria/ketonemia or DKA		3 (0.7 %)	13 (3.2 %)^b^	16 (2.0 %)
	Ketonuria or ketonemia	1 (0.2 %)	5 (1.2 %)	6 (0.7 %)
	Diabetic ketoacidosis	2 (0.4 %)	8 (2.0 %)	10 (1.2 %)
Other endocrine & metabolic diseases		6 (1.4 %)	3 (0.7 %)	9 (1.1 %)
Age-related cataract		2 (0.5 %)	6 (1.5 %)	8 (1.0 %)
Osteoarthropathy		1 (0.2 %)	4 (1.0 %)	5 (0.6 %)
Hyperosmolar hyperglycemic state		4 (1.0 %)	1 (0.2 %)	5 (0.6 %)
Hospitalization for physical examination		3 (0.7 %)	2 (0.5 %)	5 (0.6 %)
Drug-induced proteinuria or eruption		3 (0.7 %)	0 (0 %)	3 (0.4 %)
Uncategorized		14 (3.4 %)	17 (4.2 %)	31 (3.8 %)

^a^Reported as *n* (%), unless stated otherwise

^b^ *P* < 0.05

Other common reasons for admission were reported as hypoglycemia (2.9 %), neoplasms (2.8 %), and ketonuria or diabetic ketoacidosis (2.0 %). Patients were also admitted to hospital occasionally for other endocrine and metabolic disease (1.1 %), age-related cataract (1.0 %), hyperosmolar hyperglycemic syndrome (0.6 %), osteoarthropathy (0.6 %), physical examination (0.6 %), or drug-induced proteinuria or cutaneous eruption (0.4 %). Neoplastic disease was the reason given for 2.8 % of admissions, which happened more frequently in men (4.1 %) than women (1.5 %; *P* < 0.05). Moreover, ketonuria or diabetic ketoacidosis was more commonly seen in women (3.2 %) than in men (0.7 %; *P* < 0.05).

### Hospital admissions attributable to infection

There were 128 hospital admissions attributable to infections, which was the third most common reason reported for hospitalization. Specifically, these were: respiratory tract infection (44.5 %); urinary tract infection (20.3 %); gastrointestinal infection (14.8 %); and skin and soft tissue infection (10.9 %) (Table 3). Respiratory tract infection was significantly more common in men (61.4 %) than in women (31 %; *P* = 0.001), whereas urinary tract infection was more prevalent in women (29.6 %) than in men (8.8 %, *P* = 0.004).

**Table 3:** Hospital admissions attributable to infection analyzed by gender ^a^

	Men	Women	*P*
Subjects, *n*	57	71	
Respiratory tract	35 (61.4 %)	22 (31.0 %)	0.001 ^b^
Urinary tract	5 (8.8 %)	21 (29.6 %)	0.004 ^b^
Gastrointestinal tract	7 (12.3 %)	12 (16.9 %)	0.618
Skin and soft tissues	4 (7.0 %)	10 (14.1 %)	0.260
Other sites	6 (10.5 %)	6 (8.5 %)	0.765

^a^Reported as *n* (%), unless stated otherwise

^b^ *P* < 0.01

## Discussion

In this study, the most frequent reasons for hospital admission in elderly diabetic patients were the chronic complications of diabetes, hyperglycemia, and infection. Men and women differed in reasons for hospital admission.

Elderly patients with diabetes are a heterogeneous group with various functional and cognitive disabilities and underlying co-morbidities [15]. They are at increased risk of developing macrovascular and microvascular complications, suffer greater morbidity and mortality rates [7], and their functional status declines more rapidly [16], compared with their counterparts without diabetes. Also compared to men and women without diabetes, diabetic men are reportedly 4 times more likely to have difficulties related to self-care, and women are 2–3 times more likely to develop disabilities. Thus, gender differences should be considered in studies of the association between diabetes and functional impairments in the elderly [17]. In our present study, the principle reason for the hospitalization of elderly diabetic patients was chronic complications of diabetes, especially microvascular and macrovascular complications that increase with age. Such complications include nephropathy, retinopathy, neuropathy, peripheral arterial disease, and cardio-cerebrovascular complications. In our study population, diabetic nephropathy was the dominant reason for hospitalizations related to chronic complications of diabetes, with a significantly higher rate in men (14.9 %) than women (9.2 %). This is consistent with research that showed a gender difference in susceptibility to diabetic nephropathy, with women more resistant than men to the development and progression of diabetic kidney disease [18]. Several studies have found that in women hormones such as 17-β-estradiol (E2) are protective against diabetic kidney diseases [19].

Common geriatric syndromes occur frequently in older adults with diabetes that include cognitive impairment, depression, urinary incontinence, injurious falls, polypharmacy, and chronic pain [20]. Some of these syndromes have subtle presentations which are rarely identified if not specifically looked for [21]. Therefore, elderly diabetic patients have complex medical, psychosocial, and functional problems with cognitive impairment than non-diabetics [21–23]. They may lack the typical symptoms of hyperglycemia such as polyuria, polydipsia, and polyphagia [24]. In the present study, hyperglycemia or poor hyperglycemic control was the second most common reason for hospital admission. Of the patients admitted for hyperglycemia or poor hyperglycemic control, 21.8 % were asymptomatic and 36.6 % presented with atypical symptoms such as weight loss, fatigue, blurred vision, and nocturia. Frequently, these symptoms go unnoticed or are attributed to old age [25]. As a result, in 25 to 41 % of elderly individuals with diabetes, the disease is not recognized [26]. In fact, the typical symptoms of hyperglycemia are less common in elderly patients because the renal threshold for glycosuria increases with age and the thirst mechanisms are more likely to be impaired [27].

Diabetes mellitus is a chronic, progressive disorder that affects virtually every organ of the body. One of the problems associated with this condition is infection [28]. It has been reported that individuals with diabetes are at increased risk of various infection conditions [29–31] and infection-related hospitalization [32]. In the present study, infection was also a frequent cause of hospitalization, in fact, infections of the respiratory and urinary tracts together accounted for more than half of these hospital admissions. Moreover, we found that respiratory tract infection was significantly more common in men than women, whereas urinary tract infection was more frequently encountered in women. These findings may be explained by gender differences in anatomy, lifestyle, behavior, and socioeconomics [33]. In a systematic review by Falagas et al. [33] of gender differences in respiratory tract infections, men suffered more frequently and severely from respiratory tract infections compared with women, in particular lower respiratory tract infections that led to higher mortality. Gender-based differences in response to infection have also been reported in other studies, which reported that women have higher levels of plasma immunoglobulin (Ig) and are more resistant to exogenous antigens [34]. In addition, estrogens are generally immune enhancing, whereas androgens exert suppressive effects on both humoral and cellular immune responses [35].

In general, the risk of developing urinary tract infections is higher for patients with type 2 diabetes compared with those without diabetes [36]. Several factors are thought to predispose to urinary tract infections in diabetic patients, including older age, history of urinary tract infections, a longer history of diabetes, and increased HbA1c levels [30, 37]. The range of patient signs and symptoms can vary from classic to atypical in elderly patients [38]. In the elderly population with diabetes, autonomic neuropathy can reduce sensitivity and alter distensibility of the urinary bladder, leading to recurrent urinary tract infections or asymptomatic bacteriuria [30, 39]. Moreover, glycosuria enhances bacterial growth and impairs phagocytosis, which probably has a role in the increased incidence of urinary tract infections in diabetic patients, especially in the elderly. Vaginitis and renal microangiopathy, which occur more frequently in the elderly, may also be associated with urinary tract infections [39].

Diabetic ketoacidosis is a major, life-threatening hyperglycemic emergency [40]. Although the ratio of men to women afflicted with diabetes is roughly equal, women may be more likely than men to develop diabetic ketoacidosis [41]. Barski et al. [42] suggested that women with poorly controlled type 2 diabetes mellitus who receive oral hypoglycemic therapy require particular attention, as they are at high risk for metabolic decompensation and development of diabetic ketoacidosis. However, in that study hospitalized men and women with diabetic ketoacidosis were statistically similar for rates of in-hospital mortality and complications [42].

This study provides new data regarding reasons for hospitalization among elderly patients with diabetes. However, the results of the study should be generalized with caution in other geographic areas and hospitals. The study is retrospective and was performed at a single center, which led to unavoidable selection bias. Another limitation is that we did not conduct a subgroup analysis of those patients who were admitted more than once, because most of these patients were readmitted for the same reason.

## Conclusion

This retrospective study highlighted some of the characteristics of hospitalized elderly diabetic patients and the gender differences in causes of hospital admission. The most frequent reason reported for hospitalization of elderly diabetic patients was chronic complications of diabetes, seconded by hyperglycemia, and then infection.

## Abbreviations

HbA1c: Hemoglobin A1c
SPSS: Statistical package for the social sciences
